# CT-based dosiomics and radiomics model predicts radiation-induced lymphopenia in nasopharyngeal carcinoma patients

**DOI:** 10.3389/fonc.2023.1168995

**Published:** 2023-10-25

**Authors:** Qingfang Huang, Chao Yang, Jinmeng Pang, Biao Zeng, Pei Yang, Rongrong Zhou, Haijun Wu, Liangfang Shen, Rong Zhang, Fan Lou, Yi Jin, Albert Abdilim, Hekun Jin, Zijian Zhang, Xiaoxue Xie

**Affiliations:** ^1^ Department of Radiation Oncology Hunan Cancer Hospital/The Affiliated Hospital of Xiangya School of Medicine, Central South University Changsha, Hunan, China; ^2^ Key Laboratory of Translational Radiation Oncology, Department of Radiation Oncology, Hunan Cancer Hospital, Changsha, Hunan, China; ^3^ Department of Radiation Oncology, Xiangya Hospital, Central South University, Changsha, Hunan, China; ^4^ College of Physics and Electronic Science, Shandong Normal University, Jinan, China; ^5^ National Clinical Research Center for Geriatric Disorders, Xiangya Hospital, Central South University, Changsha, China

**Keywords:** radiation-induced lymphopenia, nasopharyngeal carcinoma, radiomics, dosiomics, machine learning

## Abstract

**Purpose:**

This study aims to develop and validate a model predictive for the incidence of grade 4 radiation-induced lymphopenia (G4RIL), based on dosiomics features and radiomics features from the planning CT of nasopharyngeal carcinoma (NPC) treated by radiation therapy.

**Methods:**

The dataset of 125 NPC patients treated with radiotherapy from August 2018 to March 2019 was randomly divided into two sets—an 85-sample training set and a 40-sample test set. Dosiomics features and radiomics features of the CT image within the skull bone and cervical vertebrae were extracted. A feature selection process of multiple steps was employed to identify the features that most accurately forecast the data and eliminate superfluous or insignificant ones. A support vector machine learning classifier with correction for imbalanced data was trained on the patient dataset for prediction of RIL (positive classifier for G4RIL, negative otherwise). The model’s predictive capability was gauged by gauging its sensitivity (the likelihood of a positive test being administered to patients with G4RIL) and specificity in the test set. The area beneath the ROC curve (AUC) was utilized to explore the association of characteristics with the occurrence of G4RIL.

**Results:**

Three clinical features, three dosiomics features, and three radiomics features exhibited significant correlations with G4RIL. Those features were then used for model construction. The combination model, based on nine robust features, yielded the most impressive results with an ACC value of 0.88 in the test set, while the dosiomics model, with three dosiomics features, had an ACC value of 0.82, the radiomics model, with three radiomics features, had an ACC value of 0.82, and the clinical model, with its initial features, had an ACC value of 0.6 for prediction performance.

**Conclusion:**

The findings show that radiomics and dosiomics features are correlated with the G4RIL of NPC patients. The model incorporating radiomics features and dosiomics features from planning CT can predict the incidence of G4RIL in NPC patients.

## Introduction

RT, the primary treatment for nasopharyngeal carcinoma, has been found to provide a satisfactory 5-year overall survival rate (OS) (1). Although RT is locally targeted at the tumor and damages DNA in the cells to suppress tumor growth, it unavoidably exposes normal tissues to some radiation and causes complications (2). One of the common side effects induced by RT is lymphopenia. The toxicity of radiotherapy, as evidenced by increasing evidence, has been identified as radiation-induced lymphopenia (RIL) (3) and has been reported to be a detrimental prognostic factor in those receiving radiotherapy for various solid tumors, including NPC (4–6).

The treatment strategy for cancer patients undergoing radiation therapy must take into account the issue of minimizing the occurrence of RIL. Current studies have made some efforts to explore possible factors related to RIL, including the dose–volume histogram (DVH) of lymphocyte-related organs at risk (LOARs) (7). Adults’ primary hematopoiesis site is the bone marrow, with the pelvis, cervical vertebrae, thoracic vertebrae, lumbar vertebrae, sacrum, skull, sternum, and ribs/clavicle contributing around 25%, 4%, 20%, 17%, 9%, 3%, 3%, and 9%, respectively (8). The elimination of resident lymphocytes and progenitor cells in bone marrow is likely a factor in lymphopenia. It was found that the relative volume of sternum bone marrow irradiated by more than 20 Gy could obviously affect the peripheral blood lymphocytes in patients with ESCC (9). WU et al. (10) found that there was a significant association between lymphopenia of grade 3 or higher and the radiation doses received by the thoracic vertebrae and ribs in patients with esophageal cancer who underwent neoadjuvant chemoradiotherapy. Specifically, they observed a correlation between lymphopenia and the average dose and V5-30 of the thoracic vertebrae, as well as the average dose and V5-20 of the ribs. Sini et al. (11) found a correlation between elevated BM V40 and an increased risk of acute grade 3 or late grade 2 lymphopenia in prostate cancer patients treated with whole-pelvis RT. However, no study has been conducted to identify dosimetry factors for RIL in NPC patients to date.

It has been realized that the dose–volume factors are only discrete points on the DVH curve and cannot take full advantage of the information deeply concealed in dose distributions. The 3D dose distribution’s dosiomics (dose shape) features, extracted with great optimism, surpass the restrictions of the DVH curve and uncover many of the hidden spatial features of the dose distribution (12). Dosiomics is born directly as an extension of radiomics, which refers to the automatic extraction of quantitative imaging features to develop predictive models (13). The usability of the dosiomics features’ granularity and quantity of data, in comparison to standard parameters such as DVH, DVH metrics, and visual assessment of the 3D dose distribution, could potentially be more advantageous in supporting clinical decisions. Dosiomics has been shown to be useful in predicting radiation therapy response in several studies (14, 15). However, neither radiomics nor dosiomics biomarkers for RIL prediction in NPC patients have ever been developed to date.

In this study, we first used radiomics and dosiomics analysis to predict RIL incidence in NPC patients. In the dataset of 125 NPC patients who had undergone radiotherapy, the performance of prediction models, based on dosiomics, radiomatics, clinical factors, and all other factors was assessed and compared.

## Materials and methods

**Figure 1:**
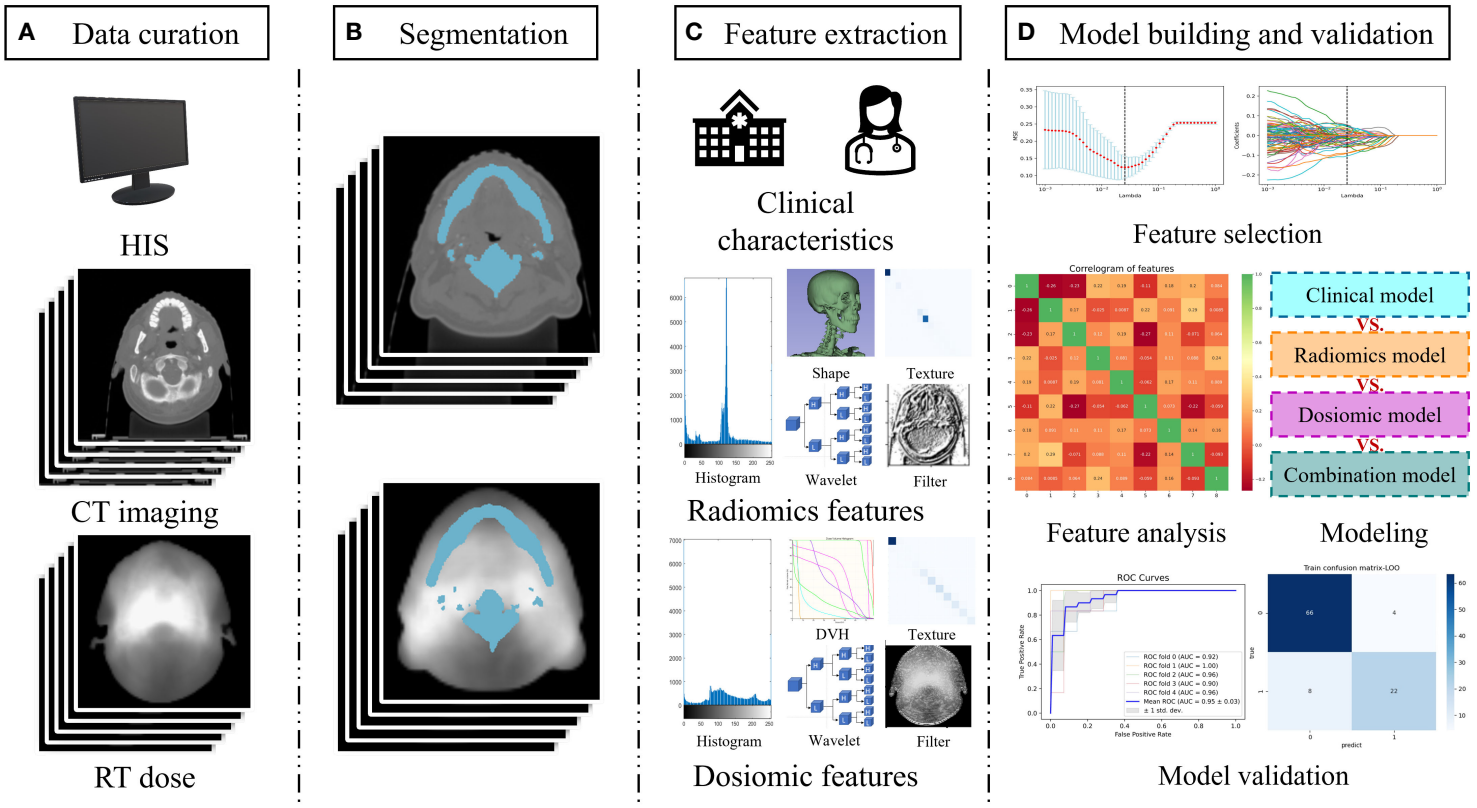
Study workflow overview. **(A)** Data acquisition; **(B)** Segmentation of the region of interest by radiologists; **(C)** Feature extraction including clinical characteristics, radiomics feature, and dosiomic feature; **(D)** Model building and validation. Abbreviation: HIS, hospital information system; DVH, dose volume histogram.

The workflow diagram for this study is shown in **Figure 1**. We extracted data from records of patients who received definitive radiation therapy (with or without chemotherapy) for biopsy-proven nasopharyngeal cancer between August 2018 and March 2019. Exclusion criteria included planned total radiation doses other than 70–74 Gy, split-course RT, simultaneous irradiation of a second primary tumor, missing records in baseline blood sample data or less than 5-week-documented ALC values during the treatment, and the unavailability of planning CT or planned biological dose maps.

### Treatment and endpoint

The Varian-600CD linear accelerator (Varian Medical Systems, Palo Alto, CA, USA) was utilized to administer volumetric-modulated arc therapy (VMAT) or intensity-modulated radiation therapy (IMRT) to all patients, with a dose of 70–74 Gy in 31–33 fractions. The RT plans were designed on Eclipse treatment planning system (TPS) (Varian Medical Systems, Palo Alto, CA). CT datasets with a 3-mm-slice thickness can be employed in either the Madison, WI, USA-based Pinnacle3 (v9.2) or the Philips Fitchburg, WI, USA-based TPS treatment planning systems. The grid size (spatial resolution of the dose distribution) in these two planning systems was 0.3 cm^3^ × 0.3 cm^3^ × 0.3 cm^3^. The beam energy for all plans was 6 MV, and the dose rate in Varian-600CD is 600 MU/min. The ultimate aim of treatment planning was to ensure a consistent and sufficient dose was delivered to the PTV and to minimize the dose to organs at risk. All patients were treated according to the principles of NPC treatment at our institute.

The endpoint of this study is the occurrence of grade 4 RT-induced lymphopenia (G4RIL), which was defined as an ALC of less than 200 cells/μL during and immediately following the course of RT.

### Delineation of ROI

This study considers the region of interest (ROI) to be the skull bone and cervical vertebrae, excluding GTV. The ROI was retrospectively delineated on plan CT with the bone windows (W2000Hu, L500Hu) and modified layer by layer with the soft tissue window (W250Hu, L50Hu). After a decade of expertise in radiation oncology, the CT images were manually segmented, and the outcomes were then evaluated by a senior radiologist. The ROIs of the CT images were all manually segmented using ITK-SNAP software (version 3.8.0; www.itksnap.org).

### Radiomics feature extraction

The incorporated CT images were normalized before extracting features. We extracted 1,734 radiomics features from ROI using PyRadiomics (Version 3.0.1, https://pyradiomics.readthedocs.io/). The original features, such as shape, first order, texture, Laplacian of Gaussian, wavelet, logarithm, gradient, square root, exponential, and 3D Local Binary Pattern, are all included in the io/matrix. Texture features include the Gray Level Co-occurrence Matrix (GLCM), Gray Level Run Length Matrix (GLRLM), Gray Level Size Zone Matrix (GLSZM), and Gray Level Dependence Matrix (GLDM).

### Dosiomics feature extraction

Normalize before extracting the dosiomics feature. After normalization, we used PyRadiomics (Version 3.0.1, https://pyradiomics.readthedocs.io/). From the dose distribution, the dosiomics features of the ROI can be extracted. A total of 1,476 dosiomics features were extracted from the ROI of the dose distribution, which contained 100 original features and 1,376 filtered features. Shape, first order, texture, Laplacian of Gaussian, wavelet, gradient, square root, logarithm, and exponential features are all extracted from the dosiomics. Texture features include GLDM, GLCM, GLRLM, and GLSZM.

### Feature selection

The selection of features was done to prevent overfitting, as the amount of extracted features is far greater than the amount of patients. In this study, we used a multistep-by-step feature selection method for the extracted radiomics features and dosiomics features. Utilizing a *t*-test to detect features with noteworthy distinctions, we initiated the feature selection process. Subsequently, the least absolute shrinkage and selection operator (LASSO) algorithm was applied to eliminate features that had regression coefficients that decreased to nothing as the penalty rose. Lastly, the variance inflation factor (VIF) was employed in the third step of feature selection to eliminate features with multicollinearity. Recursive feature elimination (RFE) based on support vector machines (SVM) is employed in the fourth step of feature selection, allowing for the assessment of feature prediction performance and the selection of features with superior prediction performance for modeling through iterative construction of the model. We used RFE to select clinical features with better predictive performance for modeling analysis in this study.

### Model construction and validation

The sample sizes of the two cohorts in this study were unbalanced, with the number of G4 RIL patients being much lower than the other cohort of G2–3 RIL patients. By utilizing the Borderline Synthetic Minority Over-Sampling Technique (SMOTE) algorithm, we augmented the G4 RIL patients, thereby achieving a more balanced sample size (16).

Before building the classification model, each feature extracted is normalized. The study builds predictive models based on SVM. We constructed a multivariate clinical model, a radiomics model, a dosiomics model, and a combination model that incorporated clinical, radiomics, and dosiomics components.

In this study, data enhancement was conducted on the training set in order to enhance the classification performance of the model. To assess the model’s performance, we split the test set into five subsets, four of which were used for training and one for testing. To validate the training set, fivefold or 10-fold cross-validation was conducted. After five repetitions of the process, the model’s performance was assessed by the mean. The 10-fold cross-validation process was comparable to the fivefold cross-validation process. We assessed the performance of each classification model by means of the receiver operating characteristic (ROC) curve, the area under the curve (AUC), accuracy (ACC), precision, sensitivity, and specificity metrics on both the training and test sets. By employing the DeLong test, we compared the statistical disparities between the various ROC curves.

### Statistical analysis

Using IBM SPSS Statistics (version 25; IBM Corporation, Armonk, NY, USA), a statistical analysis was conducted, utilizing Python (version 3.7.3, https://www.python.org) and R (https://cran.r-project.org/). The Spearman rank correlation was used to evaluate the correlation of features. The ROC curves between the different models were tested using the DeLong test, and generally, *p*-values < 0.05 were considered statistically significant.

## Results

### Patient characteristics

This study included 125 patients, with 85 in the training set and 40 in the test set. **Table 1** displays the characteristics of the patients. The training set was composed of 70 individuals with G2–3 RIL and 15 with G4 RIL, while the test set was composed of 30 individuals with G2–3 RIL and 10 with G4 RIL.

**Table 1:** Patient characteristics.

Characteristic	All-data set (*N* = 125)	Training set (*N* = 85)	Test set (*N* = 40)
	No. (%)	No. (%)	No. (%)
Age (year)			
** Median (range)**	51 (27–74)	52 (27–74)	51 (28–68)
Gender			
** Men**	92 (73.6)	63 (74.1)	29 (72.5)
** Women**	33 (26.4)	22 (25.9)	11 (27.5)
T-stage^a^			
** T1**	14 (11.2)	8 (9.4)	6 (15.0)
** T2**	37 (29.6)	26 (30.6)	11 (27.5)
** T3**	46 (36.8)	31 (36.5)	15 (37.5)
** T4**	28 (22.4)	20 (23.5)	8 (20)
N-stage^a^			
** N0**	5 (4.0)	5 (5.9)	0 (0.0)
** N1**	19 (15.2)	16 (18.8)	3 (7.5)
** N2**	78 (62.4)	51 (60.0)	27 (67.5)
** N3**	23 (18.4)	13 (15.3)	10 (25.0)
Clinical staging^a^			
** I**	1 (0.8)	1 (1.2)	0 (0.0)
** II**	10 (8)	10 (11.8)	0 (0.0)
** III**	70 (56)	45 (52.9)	25 (62.5)
** IV**	44 (35.2)	29 (34.1)	15 (37.5)
EGFR			
** Yes**	19 (15.2)	13 (15.3)	6 (15.0)
** No**	106 (84.8)	72 (84.7)	34 (85.0)
RIL grade			
** G2**	15	12	3
** G3**	85	58	27
** G4**	25	15	10

a According to the eighth edition of the International Union against Cancer/American Joint Committee on Cancer (UICC/AJCC) staging manual.

### Features selection

**Figure 2:**
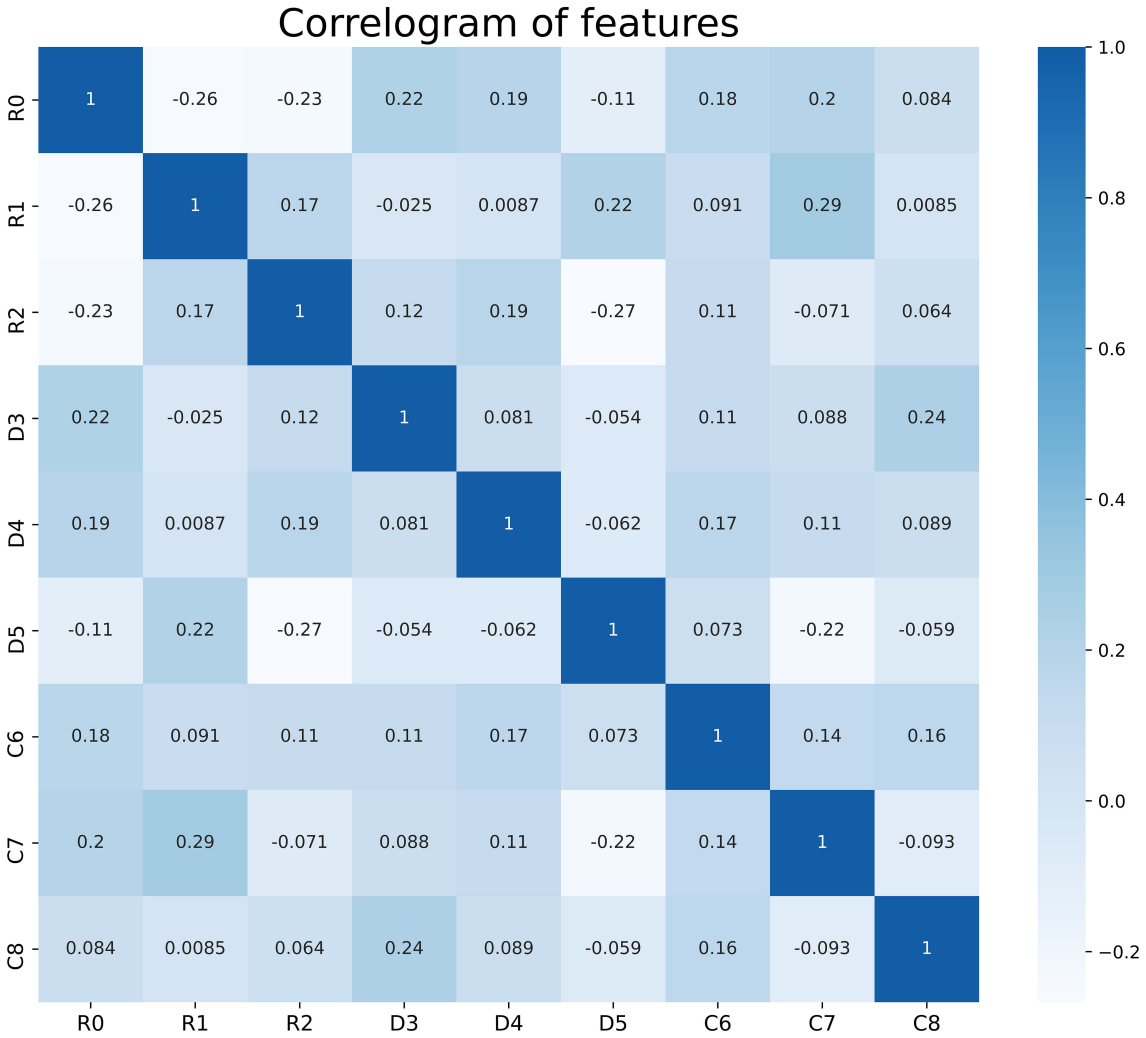
Correlation analysis of the features used in the model, there is no correlation between these features. R0- R2 are radiological features; D3- D5 are dosiomic features; C6- C8 are clinical features. R0, wavelet-LHL_glcm_Idn; R1, logarithm_glszm_Gray Level NonUniformity Normalized; R2, wavelet-HHL_glcm_Maximum Probability; D3, original_shape_Major Axis Length; D4, log-sigma-4-0-mm-3D_glszm_Small Area Emphasis; D5, wavelet-LLH_firstorder_Mean; C6, Age; C7, baseline_ALC; C8, Volume of GTVnx.

**Figure 3:**
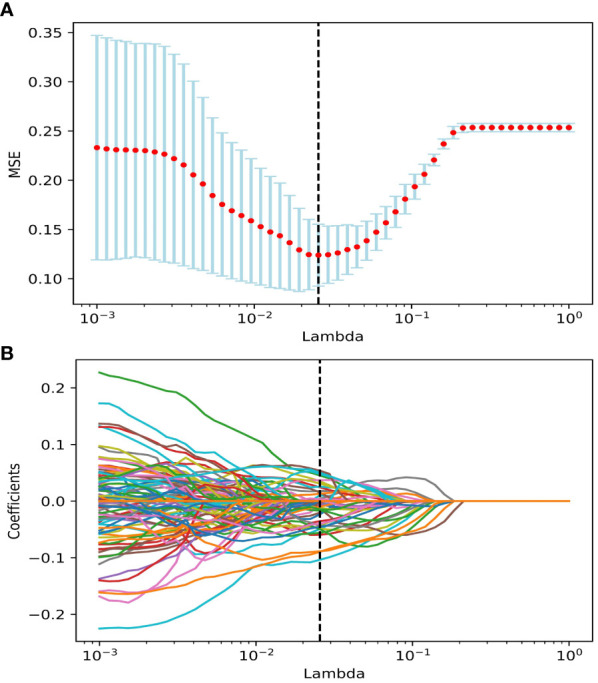
The features were selected using the LASSO regression model. **(A)** Selection of the regulation parameter lambda (λ). The vertical black dashed line defines the optimal λ at the minimum MSE. **(B)** LASSO coefficient curves of features. Vertical black dashed lines are drawn at the best lambda in **(A)**, non-zero features under the best λ are selected. Abbreviation: LASSO, the least absolute shrinkage and selection operator; MSE, Mean Squared Error.

We performed data augmentation by using the Borderline SMOTE algorithm on G4 RIL patients in the training set. After the multistep-by-step feature selection process, nine features were finally obtained for model construction. These nine features were identified as robust features, and the correlation heat map is shown in **Figure 2**, which contained three radiomics features, three dosiomics features, and three clinical features. **Figure 3** illustrates the LASSO algorithm’s selection process for features that minimize the loss function through parameter alteration.

### Development and evaluation of the model

We built a radiomics model based on radiomics features, a dosiomics model based on dosiomics features, and a clinical model based on clinical features for this study. By blending radiomics, dosiomics, and clinical characteristics, a model of amalgamation was created.

**Figure 4:**
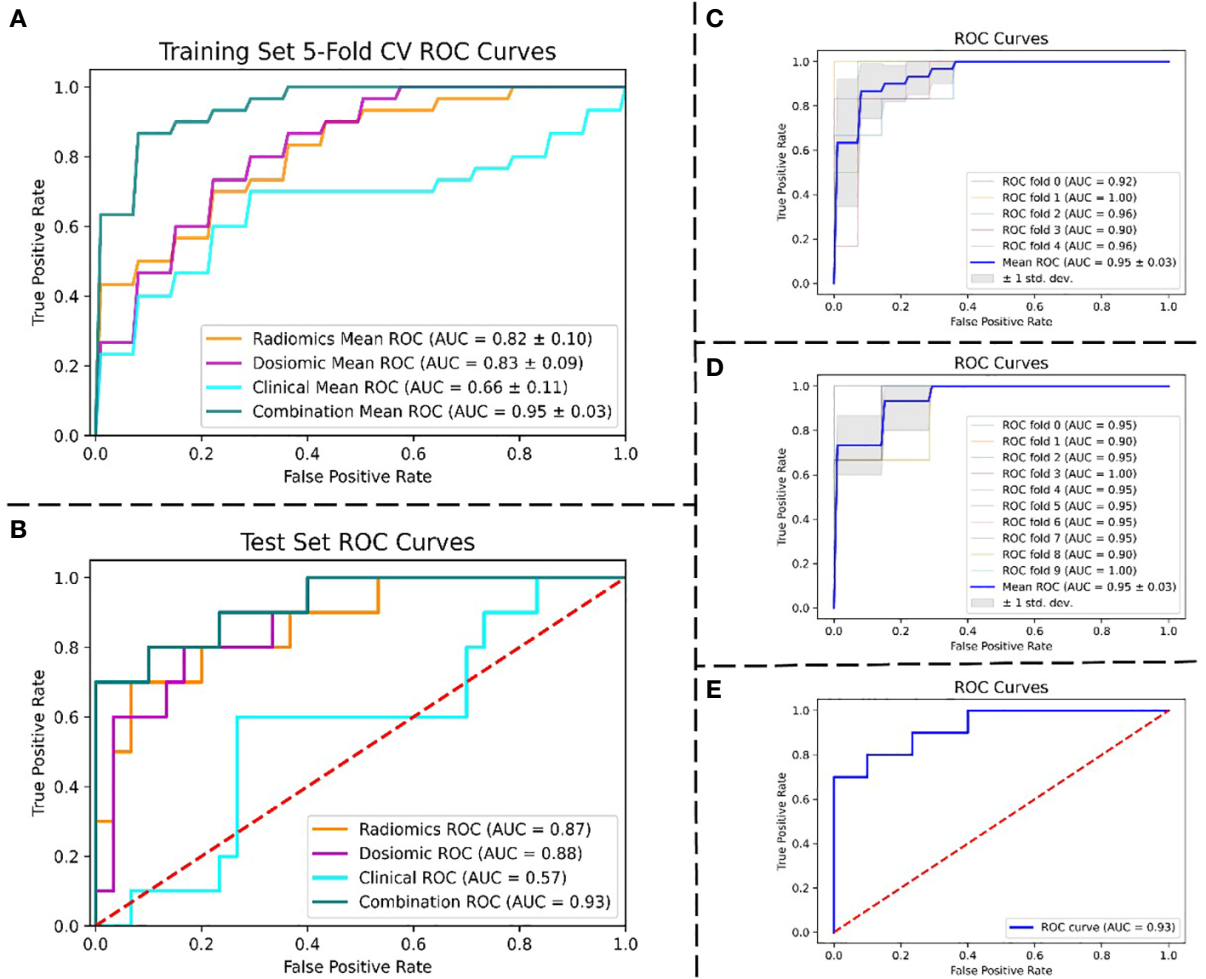
Performance of the ROC curves. **(A)** ROC curves of four models were compared using 5-fold CV in the training set. **(B)** ROC curves of four models were compared in the test set. **(C)** 5-fold CV ROC curves for the combined model in the training set. **(D)** 10-fold cross-validation ROC curves for the combined model in the training set. **(E)** ROC curve for the combined model in the test set. Abbreviation: ROC, the receiver operating characteristic; AUC, the area under the curve; CV, cross-validation.

**Figure 4** displays the ROC curves of the four models for predicting RIL in NPC. **Figure 4A** demonstrates the fivefold cross-validation in the training set, with the radiomics model having an AUC of 0.82 (95% confidence interval (CI): 0.72–0.92), the dosiomics model having 0.83 (95% CI: 0.74–0.92), the clinical model having 0.66 (95% CI: 0.55–0.77), and the combination model having 0.95 (95% CI: 0.92–0.98). The radiomics model, dosiomics model, clinical model, and combination model all had AUC values significantly higher than the clinical model (*p* < 0.05), as **Figure 4B** demonstrates. The radiomics model had an AUC of 0.87, the dosiomics model had 0.88, the clinical model had 0.57, and the combination model had 0.93. The difference between the combination model and the radiomics model was significant (*p* < 0.05). No statistically significant difference was found between the combination model and the dosiomics model (*p* = 0.09), yet the combination model still proved to be superior (AUC, 0.93 vs. 0.89; ACC, 0.88 vs. 0.82). The performance of the four prediction models was summarized in detail in **Table 2**.

**Table 2:** Performance comparison of four models: radiomics model, dosiomics model, clinical model, and combination model.

Model	Training set	Test set				
	Mean AUC (95% CI)	AUC	ACC	Precision	Sensitivity	Specificity
**Radiomics**	0.82 (95% CI, 0.72–0.92)	0.87	0.82	**1**	0.3	**1**
**Dosiomics**	0.83 (95% CI, 0.74–0.92)	0.88	0.82	0.64	0.7	0.87
**Clinical**	0.66 (95% CI, 0.55–0.77)	0.57	0.6	0.13	0.1	0.77
**Combination**	0.95 (95% CI, 0.92–0.98)	**0.93**	**0.88**	0.73	**0.8**	0.9

CI, confidence interval; AUC, the area under the receiver operating characteristic curve; ACC, accuracy. The values denoted in bold within the table signifies the best values of performance.

The best RIL prediction model was the combination model, which contained three radiomics features, three dosiomics features, and three clinical features (age, baseline_ALC, and volume of GTVnx). A combination model’s AUC of 0.95 (95% CI: 0.92–0.98) was revealed by both a fivefold and 10-fold cross-validation of the training set. The AUC of the combination model in the test set was 0.93 (accuracy: 0.88; specificity: 0.9). The combination model’s ROC curves for fivefold cross-validation (**Figure 4C**), 10-fold cross-validation (**Figure 4D**), and test set (**Figure 4E**) are depicted in **Figure 4**, and **Table 2** gives the evaluated performance of the model.

## Discussion

In the study, three clinical features, three dosiomics features, and three radiomics features were extracted from cervical vertebrae and skull bone to build prediction models for G4RIL in NPC. Using only clinical features, dosiomics features, radiomics features, and a combination of all, four models were constructed. We found that the best performance was achieved when all features were added in, and the combination model provides an expected strategic evaluation method for the radiation plans of NPC. This is the first study that has built an RIL prediction model based on dosiomics analysis.

After examining the study’s outcomes, we discovered that relying solely on clinical factors like GTVnx volume, the age of the patients, and the ALC before RT had limited predictive power for G4RIL. The AUC of the clinical model was 0.66 in the training set and 0.57 in the test set. The results suggest that more information about patients’ physiopathological characteristic and treatment process should not be omitted. Therefore, the radiomics and dosiomics methods were considered effective tools for quantitative information analysis from images and 3D RT-dose distribution in our study.

The rapid expansion of radiomics research has enabled the extraction of feature data from medical images with high throughput, and it is a noninvasive quantitative technique (17, 18). The general hypothesis of radiomics is that imaging characteristics reflect physiopathological tissue information, which is thus made accessible through quantitative features (19). Radiomics, taking radiomics features from medical images and transforming them into data that can be utilized (17, 20), is a field of study. Several studies on radiomics have shown that texture features can provide more predictive information (21–23), and some transformations may enhance texture features. In the study, three predictive radiomics features for RIL include wavelet-LHL_glcm_Idn, logarithm_glszm_gray level nonuniformity normalized, and wavelet-HHL_glcm_maximum probability. The radiomics model’s AUC, as depicted in **Figure 4**, was 0.82 in the training set and 0.87 in the test set.

Compared to traditional dosimetry analysis, dosimetry analysis demonstrated more promising results, e.g., after IMRT for head and neck cancer, locoregional recurrence has been documented (24), carbon-ion radiotherapy in skull-base chordoma has been linked to local control (25), lung cancer patients treated with radiotherapy have experienced acute-phase weight loss (26), and radiation pneumonitis has been linked to lung stereotactic body radiation therapy (27). In the study, three predictive dosiomics features for RIL include original_shape_major axis length, log-sigma-4-0-mm-3D_glszm_small area emphasis, and wavelet-LLH_firstorder_mean. As shown in **Figure 4**, the AUC of the dosiomics model was 0.83 in the training set and 0.88 in the test set.

Through the analysis of big data information from images and 3D RT dose, both radiomics and dosiomics models showed stronger predictive power than traditional clinical models. However, the robustness of the models based only on radiomics or dosiomics features in this study needs to be improved, and the error range of cross-validation is relatively large. Without a doubt, the RIL predictive models, which were based solely on radiomics or dosiomics features, were significantly enhanced in both predictive power and robustness when all features were amalgamated. The difference between the combination model and either radiomics or dosiomics models was significant (*p* < 0.05). In the training set, the AUC of the combination model was 0.95, as depicted in **Figure 4**; however, in the test set, it was 0.93. We successfully established G4RIL predictive models on NPC cancer cases by introducing dosiomics from RT three-dimensional dose distribution to an image-based radiomics strategy. This research is the first of its kind to contemplate 3D dose distribution in NPC RIL forecasting, to our knowledge.

The outcome prediction using radiomics and dosiomics analysis based on medical images and spatial dose distribution is helpful in developing clinical decision-making for the personalization of patients’ treatment. Accordingly, for NPC patients with a high predicted G4RIL risk, the therapeutic scheme may need to protect cervical vertebrae and skull bone appropriately while focusing on killing cells in the tumor area.

We have to admit that the patient dataset for this study is limited. First and foremost, the dosiomics features demonstrate good prediction ability, while the understanding of these features is still qualitative. The main reason is that the process of transforming dose distribution into GLCM, GLRLM, GLSZM, and GLDM cannot be accurately described with analytic function. Therefore, the features based on GLCM, GLRLM, GLSZM, and GLDM are not as simple and straightforward as dosimetry factors. Therefore, how to utilize the features for treatment plan design is not quite clear. In other words, currently, the dosiomics-based prediction model can only be used to evaluate an RT plan rather than help make an RT plan. It is anticipated that by gaining a more profound comprehension and accurate application of those features, this future predictive model could be used to revolutionize cancer treatment by providing clinicians with valuable tools for treatment planning, dosimetry optimization, and patient stratification. (1) The expected predictive models developed in the study can provide valuable insights into these aspects of cancer treatment. By utilizing the predictive models, clinicians can gain a better understanding of the potential outcomes of different treatment options. This information can help guide treatment planning decisions by providing insights into the likelihood of treatment success or failure. For example, if the models predict a high probability of treatment failure, clinicians may consider alternative treatment strategies or adjust the treatment plan to improve the chances of success. (2) Dosimetry optimization is another area where the future study’s findings can have a significant impact. Optimizing dosimetry involves finding the best balance between delivering an effective dose to the tumor and minimizing radiation exposure to healthy tissues. The predictive models should assist in this process by providing information on the expected response of the tumor to different radiation doses. This can help clinicians optimize the radiation dose distribution to maximize tumor control while minimizing the risk of side effects. (3) Patient stratification based on risk is an essential aspect of personalized medicine. The predictive models developed in future studies should aid in identifying patients who are at higher risk of treatment failure or experiencing severe side effects. By stratifying patients based on their individual risk profiles, clinicians can tailor treatment plans to suit each patient’s specific needs.

Another limitation that should never be overlooked is the limited sample size in this study. There are several potential strategies to expand the dataset or conduct external validation through future research to evaluate the generalizability of the predictive model, including multicenter studies, retrospective studies, multimodal datasets, data sharing, and external validation. For the multimodal dataset, other than radiation therapy dose, other parameters related to radiation therapy, such as patient’s age, gender, and pathological type, can also be considered. By collecting these multimodal data, a more comprehensive predictive model can be constructed, and the size of the dataset can be increased. By sharing the dataset with other research teams, institutions can expand the dataset through collaboration. This collaboration can be achieved through data-sharing agreements or data-sharing platforms, allowing more researchers to use the data for model validation and evaluation. For external validation, independent datasets should be used to validate the generalizability of the predictive model. These datasets can come from other research teams’ studies or publicly available clinical databases. By validating the model on different datasets, its performance and reliability in different samples can be assessed. In our further study, we will employ the above strategies to improve the reliability and generalizability of the predictive model and better evaluate the predictive effect of radiation therapy dose on radiation-related lymphocyte toxicity.

## Conclusion

Radiomics and dosiomics analyses predicting the risk of G4RIL in NPC patients were implemented for the first time, integrating CT, dose maps, and clinical features. Demonstrating that radiomics and dosiomics features can be beneficial for risk modeling of G4RIL in NPC patients in a highly conformal regime of modern radiotherapy, we still require thorough validation before they can be put into practice.

## Data availability statement

Due to further research needs, the data will not be made public within three years. After three years, the dataset may be made available upon request to the corresponding author.

## Ethics statement

The studies involving humans were approved by Ethics Committee of Xiangya Hospital, Central South University/Hunan Cancer Hospital. The studies were conducted in accordance with the local legislation and institutional requirements. Written informed consent for participation was not required from the participants or the participants’ legal guardians/next of kin in accordance with the national legislation and institutional requirements.

## Author contributions

XX and ZZ wrote the main manuscript text and CY and QH prepared figures and tables. CY did the statistical analysis and QH collected the primary data. All authors reviewed the manuscript. All authors contributed to the article and approved the submitted version.
